# PARP1 inhibition affects pleural mesothelioma cell viability and uncouples AKT/mTOR axis via SIRT1

**DOI:** 10.1111/jcmm.12000

**Published:** 2013-01-10

**Authors:** Giulia Pinton, Arcangela Gabriella Manente, Bruno Murer, Elvira Marino, Luciano Mutti, Laura Moro

**Affiliations:** a Department of Pharmaceutical Sciences, University of Piemonte Orientale A. AvogadroNovara, Italy; b Department of Anatomic Pathology, Mestre HospitalItaly; c Department of Oncology and Medicine, Vercelli HospitalItaly

**Keywords:** PARP1, Malignant pleural mesothelioma, PARP1 inhibitor, therapeutic target, AKT/mTOR axis

## Abstract

Malignant Pleural Mesothelioma (MMe) is a rare but increasingly prevalent, highly aggressive cancer with poor prognosis. The aetiology of MMe is essentially a function of previous exposure to asbestos fibres, which are considered to be an early-stage carcinogen. Asbestos is toxic to human mesothelial cells (HMCs), that activate the nuclear enzyme poly(ADP-ribose) polymerase-1 (PARP1) to repair DNA. The targeting of PARP1 is showing considerable potential for delivering selective tumour cell kill while sparing normal cells, and offers a scientifically rational clinical application. We investigated PARP1 expression in normal mesothelial and MMe tissues samples. Immunohistochemical analysis revealed low PARP1 staining in peritumoural mesothelium. As opposite, a progressive increase in epithelioid and in the most aggressive sarcomatoid MMe tissues was evident. In MMe cell lines, we correlated increased PARP1 expression to sensitivity to its inhibitor CO-338 and demonstrated that CO-338 significantly reduced cell viability as single agent and was synergistic with cis-platin. Interestingly, we described a new correlation between PARP1 and the AKT/mTOR axis regulated by SIRT1. SIRT1 has a role in the modulation of AKT activation and PARP1 has been described to be a gatekeeper for SIRT1 activity by limiting NAD+ availability. Here, we firstly demonstrate an inverse correlation between AKT acetylation and phosphorylation modulated by SIRT1 in MMe cells treated with CO-338. In conclusion, this study demonstrates that PARP1 overexpression defines increased responsiveness to its inhibition, then these results imply that a substantial fraction of patients could be candidates for therapy with PARP inhibitors.

## Introduction

Malignant Mesothelioma (MMe) is an asbestos-related tumour, with poor prognosis, refractory to current therapies, whose incidence is expected to increase dramatically in the coming decades [1]. Only a fraction of volunteers exposed to high levels of asbestos develops MMe, suggesting that additional factors and genetic predisposition may render some individuals more susceptible to asbestos carcinogenicity [2–5]. Asbestos carcinogenesis has been linked to the release of cytokines and mutagenic reactive oxygen species (ROS) from inflammatory cells.

Asbestos fibres can indirectly induce genotoxicity including base substitutions, deletions, rearrangements, insertions, sister chromatid exchanges and chromosomal aberrations, which may lead to a broad spectrum of mutations in mammalian cells [6–9]. Asbestos is cytotoxic to human mesothelial cells (HMCs). It induces HMCs programmed necrosis that involves poly(ADP-ribose) polymerase-1 (PARP1) activation, H_2_O_2_ secretion, ATP depletion, HMGB1 and TNFα release [10].

The nuclear enzyme PARP1 is a pleiotropic molecular sensor of DNA damage that exerts an important role in maintaining genomic integrity, so it represents a novel target in cancer therapy. Cells deficient in functional PARP1 display severely impaired base excision repair and genomic instability, suggesting that the enzyme may play a primary role in the cellular response to DNA damage [11–13].

The amino-terminal DNA-binding domain of PARP1 contains two zinc fingers that are important for the binding to single-strand and double-strand breaks. A third zinc finger was recently described and found to be dispensable for DNA binding, but is important for coupling damage-induced changes in the DNA-binding domain to alterations in PARP1 catalytic activity. In the central auto-modification domain, specific glutamate and lysine residues serve as acceptors of ADP-ribose moieties, thereby allowing the enzyme to polyADP-ribosylate itself. These processes are important for cell survival upon extensive DNA damage, but in normal cells the complete absence of PARP1 protein or the inhibition of PARP1 catalytic activity produces no significant growth defect.

Poly(ADP-ribose) polymerase-1 inhibition is considered as a useful therapeutic strategy for the treatment of different tumours. Inhibitors of PARP1 have been shown to enhance the cytotoxic effects of ionizing radiation and DNA damaging chemotherapy agents, such as the alkylating agents. Recent *in vivo* and *in vitro* evidences suggest that PARP1 inhibitors could be used not only as chemo/radiotherapy sensitizers, but can also act as single agents [14–18].

The PI3K/AKT/mTOR pathway has been shown to play a significant role in many functions critical to MMe generation and maintenance, including apoptotic resistance. Blockade of mTOR seems to be an effective anti-cancer strategy, even if it has been described to enhance AKT activity by feedback mechanisms involving RICTOR-mTOR activity that could induce undesirable compensatory resistance mechanisms [19].

As described in other cell models [20, 21], we show that PARP1 inhibition triggers AKT activation but disclose the first insight on the role of PARP1/SIRT1 balancing in the control of the AKT/mTOR axis providing a further rationale for the treatment of this aggressive cancer. In this study, we provide the first evidence that PARP1 is highly expressed in MMe tissues and that inhibition of PARP1 activity can be a good strategy to selectively kill MMe cells.

## Materials and methods

### Reagents and antibodies

The monoclonal antibodies specific for α-tubulin, PARP1 and the polyclonal antibodies specific for AKT, pAKT (Ser473) and acetyl lysine were from Santa Cruz Biotechnology (Santa Cruz, CA, USA). Antibodies specific for mTOR, phospho mTOR and SIRT1 were from Cell Signalling Technology Inc. (Danvers, MA, USA). The monoclonal antibody specific for poly(ADP–ribose) was from Alexis (Vinci, Fi, Italy). Antimouse and antirabbit IgG peroxidase conjugated antibodies and chemical reagents were from Sigma–Aldrich (St Louis, MO, USA). ECL was from Amersham Pharmacia Biotech (Uppsala, Sweden). Nitrocellulose membranes and protein assay kits were from Bio-Rad (Hercules, CA, USA). Culture media, sera, antibiotics and LipofectaMINE were from Invitrogen (Carlsbad, CA, USA). Cisplatin was from Ebewe Italia Srl, Rucaparib (CO-338, formerly known as AG014699 and PF-01367338) PARP1 inhibitor was firstly provided by Pfizer (New York, NY, USA) and then by Clovis Oncology Inc. (San Francisco, CA, USA).

### Immunohistochemistry

Poly(ADP-ribose) polymerase-1 protein expression levels on human tissues were assessed using an immunohistochemistry (IHC) based assay. Appropriate ethical approval was obtained from the local research ethics committees to carry out this study. Immunohistochemical stain was performed on three micron thick paraffin sections with monoclonal antibody recognizing PARP1 (Santa Cruz Biotechnology). Tissue sections were de-paraffinated according to established procedures and quenched with 3% hydrogen peroxidase for 5 min. They were then washed in running water and Tris Buffer Saline (TBS) consisting of 50 mM Tris-HCl (pH 7.6) 150 mM NaCl and 0.05% Tween 20. Heat-induced antigen retrieval was performed with a microwave oven and citrate buffer 0.01 M pH 7.0 for 40 min. at 98°C. Sections were incubated with mouse monoclonal antibody anti-PARP1 diluted 1:50 overnight at 4°C, followed by testing with a sensitive avidin-streptavidin-peroxidase technique (Biohenex, San Ramon, CA, USA). Diaminobenzidine tetrahydrocloride was used as the chromogen and sections were counterstained with haematoxylin. Distribution and intensity were considered in the semi-quantitative assessment of nuclear staining pattern as previously described [22].

### Cell cultures, treatments and transfection

The MMe derived REN cell line that was used as the principal experimental model in this investigation was provided by Dr. S.M. Albelda (University of Pennsylvania, Philadelphia, PA, USA), H2596 cell line was provided by Dr. W. Thomas (Royal College of Surgeons, Dublin, IE), primary HMCs-TERT were obtained from patients with congestive heart failure and immortalized by expression of a human telomerase subunit and the MSTO-211H cell line was obtained from the Istituto Scientifico Tumori (IST) Cell-bank, Genoa, Italy [23, 24]. Cells were cultured in RPMI medium supplemented with 10% foetal bovine serum (FBS) at 37°C in a 5% CO_2_-humidified atmosphere. For SIRT1 silencing in REN cells, we used specific oligonucleotides siRNA by QIAGEN (Hilden, Germany) and LipofectaMINE transfection reagent, as described by the manufacturer.

### Cell lysis, immunoprecipitation and immunoblot

Cells were lysed with NP-40 lysis buffer (1% NP-40, 150 mM NaCl, 50 mM Tris-HCl pH 8, 5 mM EDTA, 10 mM NaF, 10 mM Na_4_P_2_O_7_, 0.4 Na_3_VO_4_ μg/ml leupeptin, 4 μg/ml pepstatin and 0.1 Unit/ml aprotinin). Cell lysates were centrifuged at 13, 000 × g for 10 min. and the supernatants were collected and assayed for protein concentration using the Bradford protein assay reagent (Bio-Rad). For immunoprecipitation experiments, 1–2 mg of proteins were incubated with the appropriate antibody for 1 hr at 4°C in the presence of 40 μl protein A-Sepharose beads slurry (50%) per 1 ml. The beads were washed three times with 1 ml of TBS, 0.5% Triton X-100 and once with 1 ml of TBS, 0.5% Triton X-100, 0.1% SDS and the immunoprecipitates were eluted by boiling the beads in 2X Laemmli sample buffer for 5 min. Proteins were separated by SDS-PAGE under reducing conditions and transferred to nitrocellulose. Membranes were incubated with the indicated specific antibodies, and then detected with horseradish peroxidase-conjugated secondary antibodies and the chemioluminescent ECL reagent. Densitometric analysis was performed with the GS 250 Molecular Imager (Bio-Rad).

### Cell cycle analysis

For cell cycle/apoptosis analysis, 5 × 10^5^ cells per well were seeded on tissue culture plates and treated with CO-338 at different concentrations for 24 hrs at 37°C in a 5% CO_2_ atmosphere. After incubation, adherent cells were detached with trypsin (0.5% trypsin/0.1% EDTA in PBS). Detached and suspended cells were harvested in complete RPMI and centrifuged at 500 × g for 10 min., pellets were washed with PBS and fixed with ice-cold 75% ethanol at 4°C, treated with 100 mg/ml RNAse A (Sigma–Aldrich), subsequently stained with 25 μg/ml propidium iodide (Sigma–Aldrich) and then were analysed using a flow cytometer FACS (Becton Dickinson, Milan, Italy) and Modfit software (Verity Software House, Topsham, ME, USA).

### Cell viability analysis

Human mesothelial cells, REN, MSTO-211H or H2596 cells were seeded at a density of 1 × 10^4^ cells/well into six-well plates in growth medium supplemented with FBS and incubated overnight at 37°C in a humidified environment containing 5% CO_2_ to allow the cells to become adherent. After 24 hrs, cells were grown for a further 24 hrs with CO-338 at different concentrations alone or in combination with cis-platin (IC50 concentration), in complete medium. To evaluate drugs sensitivity, cells were then trypsinized and stained with Trypan blue. The number of viable cells was counted in a Burker haemocytometer within 5 min. of staining.

### Statistical and Isobologram analysis

Data are expressed as the mean ± SEM. Differences between values obtained in a population of cells treated with different experimental conditions were determined using the unpaired *t*-test. A *P*-value of <0.05 was considered statistically significant. The theoretical basis of the isobologram method has been described in previous studies [25, 26]. Based on the dose–response curves of CO-338 and cis-platin isobolograms were constructed by plotting the IC50 values of the single drug on the *x* and *y* axes, respectively, and the theoretical additive dose combination were calculated.

## Results

### PARP1 is more highly expressed in MMe cells than in HMCs and its expression correlates with response to CO-338 treatment

**Fig. 1:**
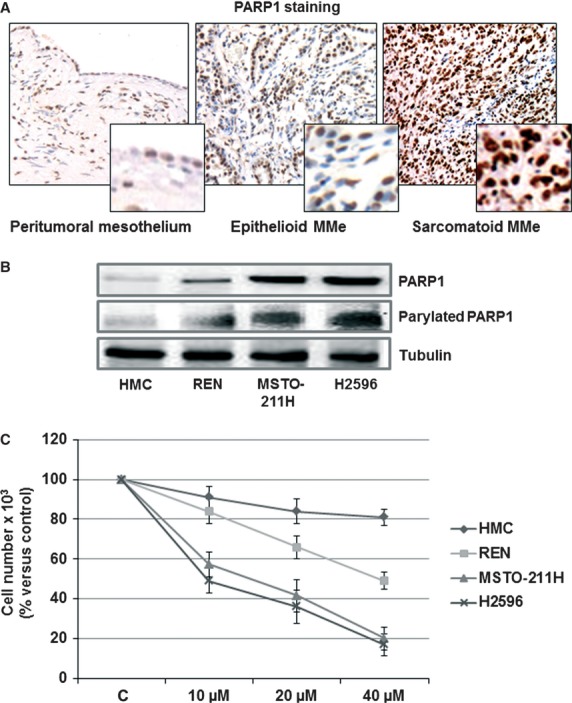
(**A**) Representative images of immunohistochemical analyses of PARP1 expression in bioptic specimens from untreated patients with confirmed pathohistological diagnosis of sarcomatoid (*n* = 30) or epithelioid (*n* = 30) MMe and their relative peritumoural mesothelium (magnification 20×). Higher magnification (60×) of some stained cells is shown in the boxed area in lower right corner. (**B**) Western blot analysis documents PARP1 expression and poly(ADP-ribosylation) in HMCs (Human mesothelial), REN (epithelioid MMe), MSTO-211H (Biphasic MMe) and H2596 (sarcomatoid) cells. Tubulin staining indicates equal loading of the proteins. Data are representative of three separate experiments. (**C**) Effects of 24 hrs treatment with different concentrations of CO-338 on HMCs, REN, MSTO-211H and H2596 cells viability. Mean ± SD of three experimental replicates.

Chemoresistance is the main obstacle of treatment of MMe [27]. We supposed that this could be related to a higher level of PARP1-mediated DNA repair. To test our hypothesis, we performed *in vivo* and *in vitro* studies. By immunohistochemical analysis, we evaluated PARP1 expression in a well-defined cohort of patients (70 patients of whom 52 males and 18 females with a median age at diagnosis of 63 years) (Table 1) with confirmed patho-histological diagnosis of sarcomatoid (*n* = 30), biphasic (*n* = 10) or epithelioid (*n* = 30) MMe and their relative peritumoural mesothelium (magnification 20×). Intense nuclear PARP1 staining (3+ in 67–100% cells) was observed in all sarcomatoid and in the sarcomatoid component of the biphasic MMe samples, whereas the expression of the protein was progressively reduced (2+, 1+ in 67–100% cells) in epithelioid MMe tissues and in peritumoural mesothelium. A representative image of PARP1 staining by immunohistochemistry in normal and MMe tissues is reported in Figure 1A.

**Table 1:** Main volunteer characteristics at diagnosis (*n* = 70)

Male	52
Female	18
Age at diagnosis, y, median (range)	63 (32–82)
Histological type, *n* (%)	
Epithelioid	30 (43%)
Biphasic	10 (14%)
Sarcomatoid	30 (43%)
Stage, *n* (%)	
T2	22 (32%)
T3	19 (27%)
T4	29 (41%)
Survival, months (range)	
Epithelioid	28.4 (4–77)
Biphasic	11.9 (1–44)
Sarcomatoid	9.6 (6–18)

Data obtained *in vitro* were comparable to those obtained *in vivo*, in fact, Western blot analysis of H2596 (sarcomatoid MMe), MSTO-211H (biphasic MMe), REN (epithelioid MMe) and HMCs (human mesothelial) cells revealed an increase in PARP1 expression and auto-modification related to tumour histotype (Fig. 1B). On the basis of these observations, we decided to test if these cells presented a different sensitivity to PARP inhibition. In Figure 1C, we show that CO-338, a PARP1 inhibitor, significantly reduced the viability of all MMe cells in a dose-dependent manner. The sensitivity of HMCs and MMe cells to CO-338 was positively related to PARP1 expression: HMCs being the less sensitive one and the biphasic MSTO-211H and the sarcomatoid H2596 cells being the most sensitive. In fact, HMCs that, as shown, express low levels of PARP1, were poorly responsive to CO-338, whereas it was effective in REN and even more in MSTO-211H and H2596 cells (IC50 at 24 hrs of treatment were 37, 16 and 14 μM respectively).

### HMCs acquire CO-338 sensitivity when PARP1 is activated by Hydrogen Peroxide treatment

**Fig. 2:**
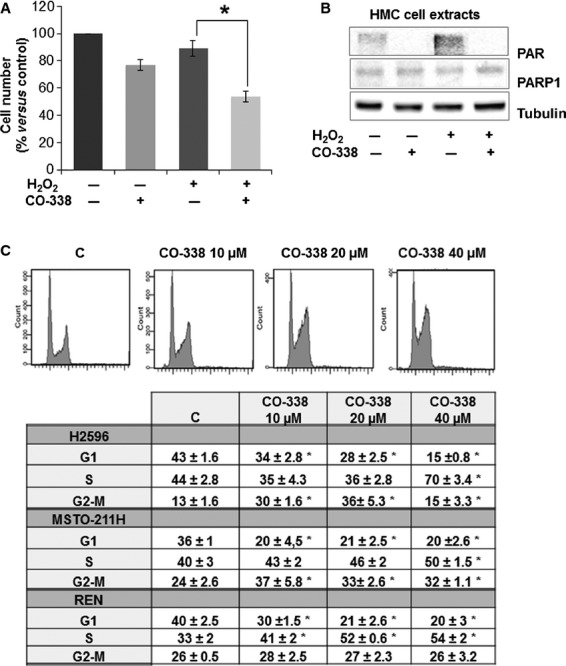
(**A**) Effects of 24 hrs treatment with 40 μM CO-338 and/or 25 μM Hydrogen Peroxide on HMCs viability. Mean ± SD of three experimental replicates. *P < 0.05. (**B**) Western blot analysis that documents PARP1 expression and poly(ADP-ribosylation) in HMCs treated with 40 μM CO-338 and/or 1 mM Hydrogen Peroxide for 10 min. Data are representative of three separate experiments. Tubulin staining indicates equal loading of the proteins. (**C**) H2596, MSTO-211H and REN cells were treated with different concentrations of CO-338 for 24 hrs. After treatments, cells were stained with propidium iodide as described in ‘Materials and Methods’ and analysed for cellular DNA content by flow cytometry. Exemplificative histograms that plot cell count *versus* DNA content are reported for each treatment. Data reported in the bottom graphs represent mean of the percentage of cells in each phase of the cell cycle (*n* = 3). Statistical analysis of means ± SD indicated that differences in cell cycle phase distribution were significant (*P* ≤ 0,05) at all CO-338 concentrations.

We initially assumed that HMCs were insensitive to CO-338 treatment because PARP1 was poorly expressed and activated. To clarify this evidence finding, we demonstrated that HMCs after insult with H_2_O_2_ (Hydrogen Peroxide) were more sensitive to CO-338 than untreated cells. HMCs were exposed to 25 μM H_2_O_2_ for 24 hrs and cytotoxicity was determined through a cell viability assay (Fig. 2A). Western blot analysis was also performed to assess PARP1 poly(ADP-ribosylation) following exposure to H_2_O_2_ (Fig. 2B). We confirmed that treatment with CO-338 completely abolished H_2_O_2_ induced PARP1 auto-modification.

### CO-338 inhibits MMe cell growth causing G2/M phase cell cycle arrest

We have shown that treatment of MMe cells with different concentrations of CO-338 significantly reduced the viability of MMe cells, so we decided to investigate more deeply on cell cycle distribution by cytofluorimetric analysis. We observed that 24 hrs treatment of REN, MSTO-211H and H2596 cells with increasing concentrations of PARP1 inhibitor caused cell cycle arrest with a statistically significant progressive accumulation of cells in the G2/M phases of cell cycle (Fig. 2C). No significant differences between the cell lines tested were observed.

### Inhibition of PARP1 activity leads to AKT de-acetylation and phosphorylation, but uncouples mTOR signalling in MMe cells

**Fig. 3:**
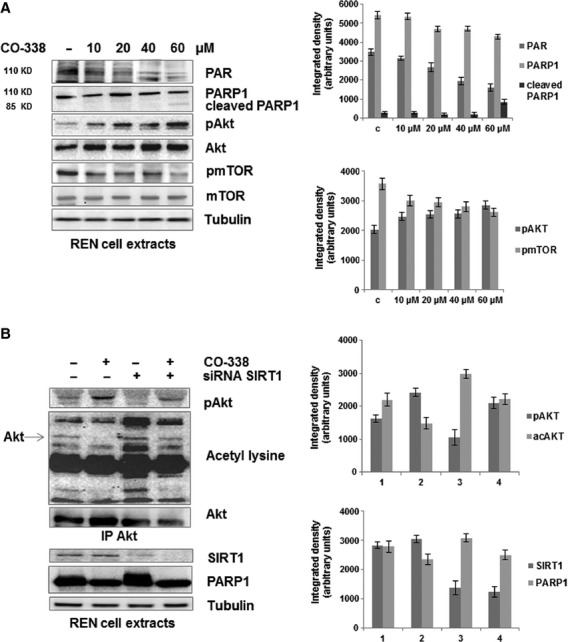
(**A**) Representative Western blot analysis that documents the reduction in PARP1 poly(ADP-ribosylation), the increase of AKT and the reduction of mTOR phosphorylation in response to treatment with different concentrations of CO-338 (24 hrs) in REN cells (similar results were obtained in H2596 and MSTO-211H cells). Bar graphs show relative densitometry. (**B**) Representative immunoprecipitation experiment that documents AKT phosphorylation, acetylation and its interaction with other acetylated proteins in REN cells in silenced for *SIRT1* (as documented in the lower panel) and treated 24 hrs with 40 μM CO-338. Tubulin staining indicates equal loading of the proteins. Data are representative of three separate experiments. Bar graphs show relative densitometry.

To verify the efficacy of CO-338 on PARP1 biological activity, MSTO-211H and REN, MMe cells were exposed to different concentrations of compound and then Western blot analysis was performed to check PARP1 auto-modification. The results, shown in Figure 3A and B, document the significant reduction in PARP1 poly(ADP-ribosylation) in a dose-dependent manner. We also observed that the highest concentrations of inhibitor lead to PARP1 cleavage indicating, at these concentrations, CO-338 induced apoptosis.

In various tissues, it has been shown that the PARP1 inhibition leads to phosphorylation and thus activation of AKT, but the underlying mechanism still remains unknown. In our experiments, we observed that AKT phosphorylation is directly correlated with PARP1 inhibition. Despite AKT activation, CO-338 treatment resulted in a reduction in mTOR phosphorylation, so this pathway was unable to counteract CO-338 toxicity in MMe cells. A role for SIRT1, which is activated as a consequence of PARP1 inhibition [28], both in AKT activation and in mTOR inhibition, has been well described [29]. So we decided to explore the role of SIRT1 in the control of the balancing between acetylation and phosphorylation of AKT upon PARP1 inhibition. As demonstrated by an exemplar immunoprecipitation experiment, reported in Figure 3C, in basal conditions AKT is in part acetylated and in part phosphorylated and became highly phosphorylated and completely de-acetylated upon PARP1 inhibition. SIRT1 silencing resulted in a more evident AKT acetylation that was reduced in the presence of CO-338. Moreover, interestingly, SIRT1 silencing and CO-338 treatment resulted in different acetylation status of AKT interactors that remain to be identified.

### CO-338 enhances response to cisplatin of MMe cells

**Fig. 4:**
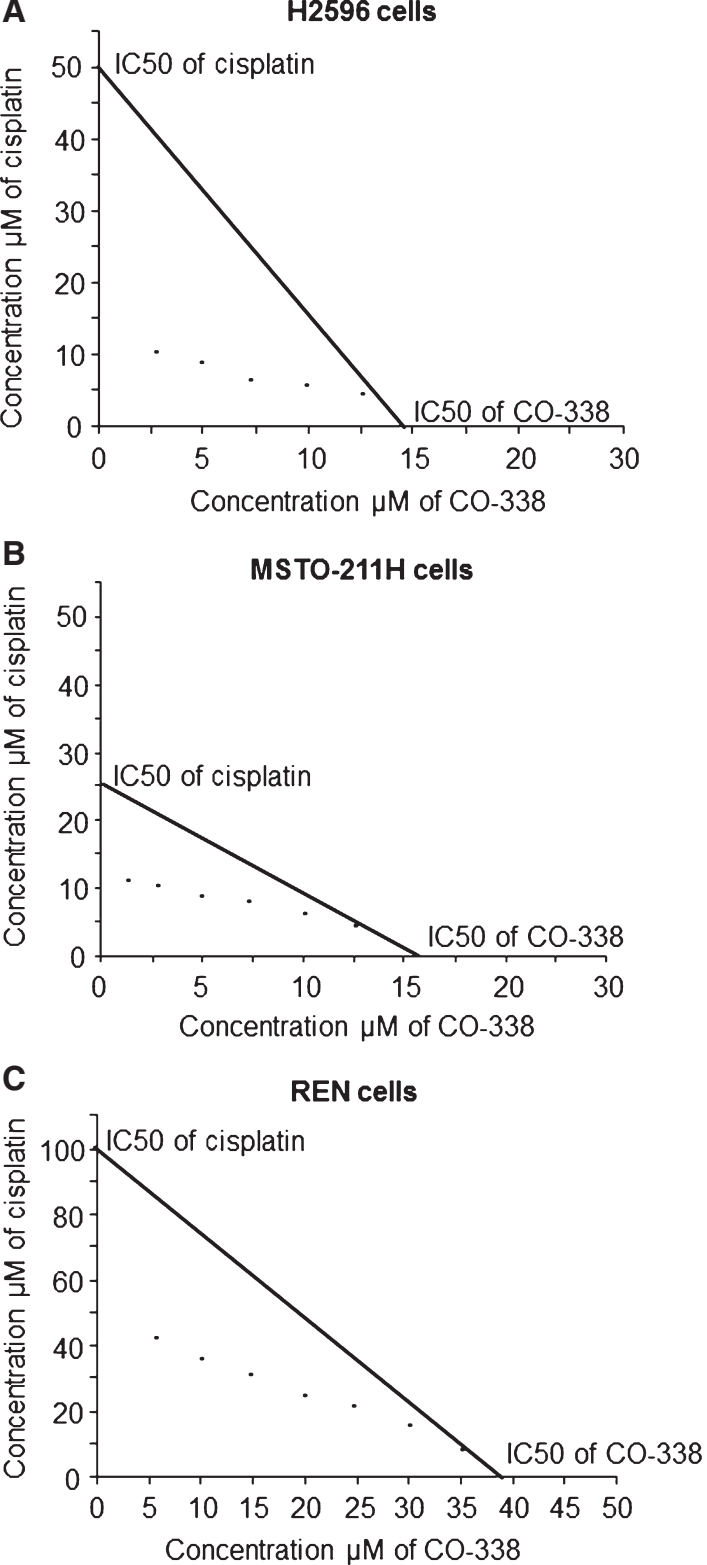
(**A–C**) Isobologram plots of the interactions between CO-338 and cisplatin on H2596, MSTO-211H and REN cells. Cells (5 × 10^4^) were plated in complete medium and allowed to adhere to the surface overnight. The plating medium was removed and replaced with medium containing various concentrations of CO-338 and the IC50 concentrations of cisplatin. To determine the nature of the interaction between CO-338 and cis-platin, we counted surviving cells following 24 hrs of drug incubation. The diagonal line represents the isoeffect line of additivity. Each point is the mean determined from experiments performed in triplicate.

Platinum drugs induce inter- and intra-strand cross-links that are removed by nucleotide excision repair. Because PARP1 participates in nucleotide excision repair [30], synergy between PARP1 inhibitors and platinum drugs is expected [31, 32]. Moreover, PARP1 has been reported to bind to DNA damaged by platinum compounds, suggesting a direct role of PARP1 in the repair of such damage [33]. Accordingly, several studies suggest that PARP1 inhibitors potentiate the effect of platinum compounds. To test interactions between PARP1 inhibition and platinum, currently used in the first line MMe therapy, we examined data from dose–response curves by isobologram analysis. We chose to evaluate anti-proliferative effects using the IC50 for single drug effects that were determined experimentally, using the data from the dose–response experiments as a starting point. CO-338 (5–35 μM for REN, 1.25–15 μM for MSTO-211H and H2596 cells) and cisplatin (IC50 was 100 μM in REN, 25 μM in MSTO-211H and in H2596 cells) were administered simultaneously for 24 hrs to cells in culture. At the end of incubation cells were trypsinized and counted. Representative isobolograms in Figure 4A and B indicate that the combination of CO-338 and cisplatin displayed strong synergistic cytotoxicity in all MMe cells across a broad range of concentrations.

## Discussion

The mechanisms of asbestos carcinogenicity are not fully understood. During the long latency period of MMe, many pathogenetic events may occur that can contribute to MMe development. The deposition of asbestos in the lung and pleura causes chronic inflammation that may lead to reactive mesothelium hyperplasia and/or transformation. Asbestos causes DNA strand breaks mediated by iron-catalysed free radicals. In addition, by causing the release of reactive oxygen species (ROS) and reactive nitrogen species (RNS), asbestos fibres can indirectly induce a broad spectrum of DNA mutations. After the induction of certain types of DNA damage, including nicks and DNA double-strand breaks, PARP1 is rapidly recruited to the altered DNA and its catalytic activity increases 10- to 500-fold, resulting in the synthesis of protein-conjugated long branched poly(ADP ribose) chains 15–30 sec. after damage [12].

It has been described that PARP1 protein expression is significantly increased in circulating lymphocytes of asbestos-exposed volunteers [34] and that asbestos-exposed human mesothelium cells secrete H_2_O_2_, activate PARP1, deplete ATP and release cytokines.

A transient inhibition of DNA repair using potent PARP1 inhibitors could improve the efficacy of cancer treatments, in fact, PARP1 inhibition is considered as a useful therapeutic strategy for the treatment of a wide range of tumours. Inhibitors of PARP1 have been shown to enhance the cytotoxic effects of ionizing radiation and DNA damaging chemotherapy agents, such as alkylating agents and topoisomerase I or II inhibitors.

Our findings identify that PARP1 expression in MMe correlates with histological type. Immunohistochemical analysis revealed low PARP1 staining in peritumoural mesothelium and a progressive increase in epithelioid and in the most aggressive sarcomatoid MMe; the same data were confirmed by Western blot analysis on HMCs and MMe derived cell lines. We demonstrated that MSTO-211H and H2596 cells, derived from biphasic and sarcomatoid MMe, respectively, although ordinarily chemo-resistant, are more sensitive to PARP1 inhibitor CO-338 than the epithelioid REN and normal mesothelial cells.

**Fig. 5:**
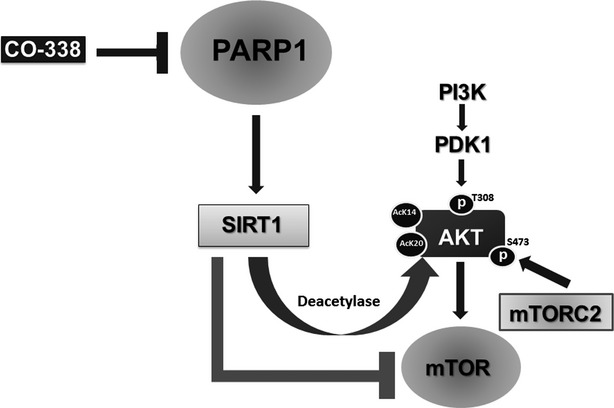
Here, we demonstrate an inverse correlation between AKT acetylation and phosphorylation modulated by SIRT1 in MMe cells treated with PARP1 inhibitor. The pharmacologic inhibition of PARP1 increases NAD+ content, SIRT1 activation and AKT de-acetylation. Moreover, we hypothesize a role for SIRT1 in mTOR down-regulation, through interaction with TSC1/TSC2, the main mTOR inhibitory complex. Blockade of mTOR, can also lead to a rebound upstream phosphorylation of AKT because mTORC2 complex is preferentially formed than mTORC1, but this doesn't seem limit the effects of mTOR inhibition.

Different studies, in cancer models, suggest that PARP1 inhibition increases AKT phosphorylation, and its inhibition is necessary to reduce the capability of cancer cells to repair DNA damage and survive after insults [35, 36]. Very interestingly, in our cancer cells, AKT activation related to PARP1 inhibition was unable to modulate pro-survival signals, probably because the downstream pathway was interrupted at the level of its effector mTOR. The pharmacologic inhibition of PARP1 *in vitro* and *in vivo* has been described to increase NAD+ content and SIRT1 activation, indicating that PARP1 is a gatekeeper for SIRT1 activity by limiting NAD+ availability. Recent data have clearly demonstrated a role of SIRT1 in the modulation of AKT activation [29]. Here, we firstly demonstrate an inverse correlation between AKT acetylation and phosphorylation modulated by SIRT1 in MMe cells treated with PARP1 inhibitor. Moreover, we hypothesize a role for SIRT1 in the down-regulation of mTOR, in fact it has been reported that SIRT1 is able to modulate mTOR phosphorylation through interaction with TSC1/TSC2, the main mTOR inhibitory complex [37, 38]. mTOR has been found to mediate survival, through either of its two downstream targets, S6K or 4E-BP1/eIF4E [39–41], suggesting that blockade of mTOR would be highly beneficial. Blockade of mTOR, however, can also lead to a rebound upstream activation of AKT. On the basis of these data, we propose a model where negative regulation of mTOR signalling by SIRT1 activation, as a consequence of PARP1 inhibition, triggers AKT phosphorylation as mTORC2 complex is preferentially formed than mTORC1, but this does not seem to limit the effects of mTOR inhibition. Future studies are needed for insight into the exact mechanism of SIRT1's regulation (Fig. 5).

It has been described that PARP1 binds to DNA damage induced by platinum compounds, suggesting a direct role of PARP1 in the repair of such damage and a synergy between PARP1 inhibitors and platinum drugs is expected. Accordingly, by experiment of cell viability, we demonstrated that in MMe cells the combined treatment of cisplatin and CO-338 resulted in augmented cell death. It has been recently published that reduced *BRCA1* expression correlates with a better outcome following platinum chemotherapy, and clinical trials report on significant anti-tumour activity following PARP inhibitor treatment in *BRCA1*-deficient patients. Loss of BRCA1 protein expression has been recently identified in 38.9% of MMe tissue samples with the highest percentage of BRCA1 immuno-negativity observed in the sarcomatoid MPM tumours [42]. We would therefore predict that MMe tumours lacking expression of *BRCA1* might also represent a molecularly defined subgroup of tumours with sensitivity to PARP1 inhibition. In conclusion, our results clearly show how both PARP1 and SIRT1 affect critical cellular pathways involved in MMe progression and offer a model of a regulatory inter-relationship between these proteins. These data could be helpful for designing new effective therapeutic strategies.
